# Ethno-botanical study of the African star apple (*Chrysophyllum albidum* G. Don) in the Southern Benin (West Africa)

**DOI:** 10.1186/1746-4269-8-40

**Published:** 2012-10-09

**Authors:** Laurent G Houessou, Toussaint O Lougbegnon, François GH Gbesso, Lisette ES Anagonou, Brice Sinsin

**Affiliations:** 1 Laboratory of Applied Ecology, Faculty of Agronomic Sciences, University of Abomey-Calavi, Cotonou, 01 PO BOX 526, Benin; 2 Department of Geography, Faculty of Letter, Arts and Human Sciences University of Abomey-Calavi, Cotonou, Benin; 3 National High School of Technical and Agronomical Sciences, University of Abomey-Calavi, Abomey-Calavi, PO Box 1967, Benin

**Keywords:** Benin, Ethnobotanical knowledge variation, Use category, Underutilized species

## Abstract

**Background:**

In addition to plant species biology and ecology, understanding the folk knowledge systems related to the use of plant species and how this knowledge system influences the conservation of plant species is an important issue in the implementation of sustainable strategies of biodiversity conservation programs. This study aimed at providing information on the use and local knowledge variation on *Chrysophyllum albidum* G. Don a multipurpose tree species widely used in southern Benin.

**Methods:**

Data was collected through 210 structured interviews. Informants were randomly selected from ten villages. The fidelity level and use value of different plant parts of *C. albidum* were estimated. The variation in ethnobotanical knowledge was assessed by comparing the use value between ethnic, gender and age groups. In order to assess the use pattern of the different plant parts in folk medicine, a correspondence analysis was carried out on the frequency citation of plant parts.

**Results:**

Four categories of use (food, medicine, firewood and timber) were recorded for *C. albidum*. With respect to the different plant parts, the fleshy pulp of the African star apple fruit showed high consensus degree as food among the informants. Fifteen diseases were reported to be treated by the different parts of *C. albidum* in the region. Correspondence analysis revealed the specificity of each part in disease treatment. There was no significant difference among ethnic groups regarding the ethno-botanical use value of *C. albidum*. However, significant difference existed between genders and among age groups regarding the knowledge of the medical properties of this species.

**Conclusions:**

*C. albidum* is well integrated in the traditional agroforestry system of the southern Benin. Despite its multipurpose character, this species remains underutilized in the region. Considering the current threat of habitat degradation, action is needed in order to ensure the long term survival of the species and local communities’ livelihoods.

## Background

Over the world, people rely on plant species for food, medicine, fodder and wood uses [1,2]. Among the plant species, the multipurpose species significantly contribute to livelihood enhancing of local populations [3-5]. Unfortunately, most of these multipurpose species are facing a decline of their populations due to the growing demand of non timber forest products (NTFPs) for household consumption as well as for local, regional and international trade [6-8]. Therefore, there is a need to assess the use pattern of these species by local populations in order to define a sustainable participatory conservation strategy for them. In this light, we focused on the use pattern of the important multipurpose species of *Chrysophyllum albidum* G. Don, in southern Benin.

In Benin, the African star apple *Chrysophyllum albidum* (Sapotaceaea) occurs on ferallitic soils [9]. *C. albidum* is a lowland rain forest tree species which can reach 25 to 37 m in height at maturity with a girth varying from 1.5 to 2 m [10]. Despite the role of ecological barrier the Dahomey Gap played in the distribution of many evergreen rain forests species in Western Africa [11], *Chrysophyllum albidum* is present in Benin.

*C. albidum* is highly used and appreciated in southern Benin, where it is called *azongogwe* or *azonbobwe* in local language "Fon, Goun" and *azonvivo, azonvovwe or azonbebi* in local language "Aïzo" [12]. Nowadays, in Benin, *C. albidum* is considered as vulnerable and its habitat seems to be restricted to traditional agroforestry systems or remnant semi-evergreen rain forest stands often protected for religious reasons [13,14].

Previous studies on *C. albidum* in western Africa reported the importance of the species for local community livelihood improving and its potentiality for food industries. For instance, the physical, chemical and nutritional characterization of *C. albidum* fruits have shown a high industrial potential [15-17]. Some ethnobotanical studies on NTFPs species have mentioned *C. albidum* as used by local communities for medicinal and food purpose [18,19].

Despite its importance, in Benin *C. albidum* is poorly investigated and this species was mentioned in the group of wild fruit tree species which need more detailed scientific information regarding their use pattern, ecology and reproduction biology in order to define a better conservation strategy [12]. Therefore, this study intended to fulfill this gap by gathering information on the use of this species in Benin.

Most studies on ethnobotanical knowledge have concluded that there is an unequal indigenous knowledge and perception of plant use among local populations related to differences in ecological regions, genders, age, ethnicity, profession, religion, cultural beliefs, and abundance and usefulness of the species being investigated [5,20-23]. Such information is relevant to ensure a sustainable and efficient implementation of future management schemes for plant species conservation [24,25]. Therefore, in this study, we also assessed the differences in local knowledge related to gender, ages and ethnic groups for *C. albidum*.

## Methods

### Study area

**Figure 1:**
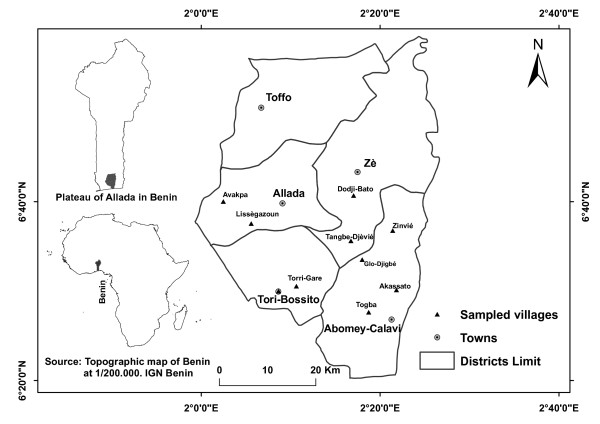
Map of the study area, with the sampled villages for survey.

The study was carried out on the ‘Plateau of Allada’ which includes five administrative districts in the Southern Benin. It geographically spans between 2°00’ to 2°30’ longitude East and 6°20’ to 6°50’ latitude North (Figure 1). The region is characterized by a bi-modal climatic regime with two rainy seasons (one long from mid March to mid July and one short from mid September to mid November) alternating with two dry seasons (one large from mid November to mid March and one short from mid July to mid September). The annual rainfall ranges from 1100 to 1300 mm with 82 to 122 rain days. The mean temperature is 27°C. February-April are the driest months while July-September are the coolest one [26]. Overall, the dominant soils in the ‘Plateau of Allada’ are ferrallitic [26]. The native vegetation is a semi-deciduous forest which has been converted (in almost its totality) in a mosaic of traditional agroforestry systems (fallows, fields and orchard) and human settlements where endogenous, cultivated and exotic plant species co-occur [27]. The population in this area is a multi-ethnic with the dominance of Aizo and Fon ethnic groups [28]. Agriculture and non-timber forest products exploitation and commercialization remain the main economic activities of the population in this area [29]. 

### Data collection

**Figure 2:**
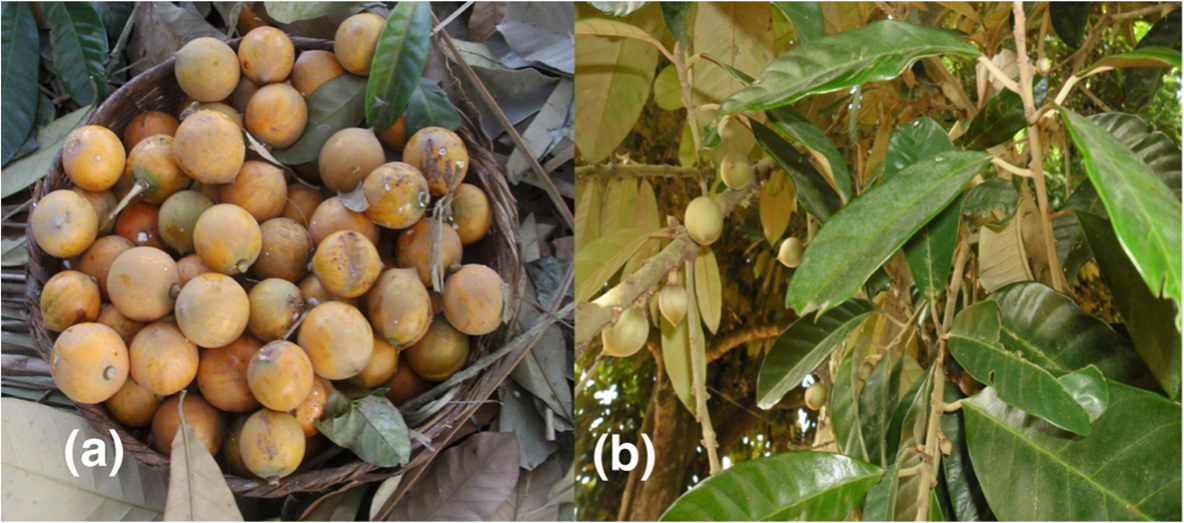
**Fruits and leaves of*Chrysophyllum albidum.*a** = Fruits of *C. albidum*, **b** = Leaves of *C. albidum.*

Preliminary investigations were carried out to determine: (i) the adequate sample size for the ethnobotanical survey, and (ii) the villages where *C. albidum* was common. For the preliminary investigations, twenty five people were randomly sampled. They were asked whether they knew and used *C. albidum* based on a picture of the species and its description (Figure 2). Moreover, they were asked if they knew where *C. albidum* was commonly found in the “Plateau of Allada”. Since 84% of the preliminary sampled population recognized and used *C. albidum*, we determined the sampled population size for this study using the formula of Dagnelie [30]:

(1) $$N=\frac{U_{\left(1-\alpha /2\right)}^{2}xP\left(1-P\right)}{d^{2}}$$

where **P** = frequency of persons knowing the species from the preliminary survey (0.84), U_1-α/2_= 1.96 (normal distribution, α = 0.05) and **d** is the expected error margin of any parameter to be computed, which we fixed here at 0.05. Therefore the sample size used for the full survey was 210. Informants were randomly chosen from ten villages where *C. albidum* was common. Table 1 summarizes the sample size of people surveyed by ethnic group, gender and age category. 

**Table 1:** Socio-demographic characteristics of the sampled population

**Ethnic group**	**Gender**		**Age**			**Total per ethnic group**
	**Men**	**Women**	**≤30 years**	**30 < years ≤ 60**	**> 60 years**	
Fon	48	42	41	22	30	90
Aïzo	55	65	30	45	42	120

Structured interviews were undertaken using a questionnaire. Questions asked during the interviews were related to (i)- the plant parts used, (ii)- the different uses of the species, (iii)- the use level of the species for each use types (e.g., food, medicine) through a coding system: 3 = highly used, 2 = fairly used, 1 = weakly used, 0 = not used, (iv)- the medicinal use of the species and, (v)- socio-cultural consideration related to the species use and conservation.

### Data analysis

The following parameters were estimated:

Fidelity level (FL)

The use frequency for the various use categories of the species and for the different plant parts was computed followed Friedman *et al.*[31] as:

(2) $$FL=\frac{S}{N}$$

where S is the number of informants who gave a positive answer to the use of *C. albidum* for a given use category. It also represents the number of informants who had positive answer to the use of a plant part (fruit, leave, bark, root, etc.) in a given category. N is the total number of informants.

Ethno-botanic use value (UV)

The ethno-botanic use value was determined to assess the importance of *C. albidum* plant parts for each ethnic age and gender group. The ethno-botanic use value was calculated following the formula of Philips and Gentry [32]. Data were arranged per use category (k) and the ethno-botanic use value (UV) in each category was computed as the mean score given by all the informants in the considered category;

(3) $$UV_{k}=\frac{1}{n}\sum_{p=1}^{n}s$$

Where **'s'** is the score attributed to *C. albidum* by the informants with respect to the use categories, **'k'** and **'n'** the number of informants. UV_k_ ranges from 0 (the species is not used for that purpose/category) to 3 (the species is reported to be highly used for that purpose/category by all the informants).

Finally, the overall ethno-botanical use value of the species was determined for each ethnic, age and gender group as:

(4) $$OUV=\sum_{i=1}^{k}UV_{k}$$

Where **'k'** is the number of use categories, UV_k_ is the estimated ethno-botanic use value of the species in the use category **'k'** for each ethnic, age and gender group.

The normality and homogeneity of the use value were checked and non parametric tests were performed to assess significant differences related to gender, age and ethnic group. Chi-sq test was used to determine whether there was statistic difference in the species fruit taste among ethnic group, gender or age. In order to assess the use pattern of the plant parts in folk medicine, correspondence analysis was carried out on the frequency of citation of the different plant parts in ailment treatment.

## Results

### Use categories of *C. albidum*

**Figure 3:**
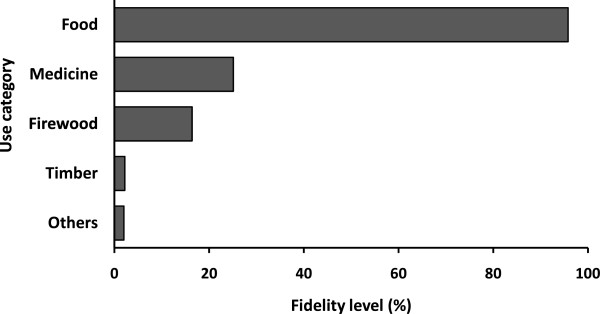
**Fidelity level use of each category of*Chrysophyllum albidum*.**

*C. albidum* was widely used by local populations for many purposes (Figure 3). Four use categories namely food, medicine, firewood and timber were recorded for *C. albidum*. Food purpose represented the most dominant category. About 95.8% of the informants exploited the species for its fruits whereas 25.1% and 16.4% respectively exploited it for medicinal and firewood purposes. The species was less used as timber (2.2% of the informants). Other used categories such as fodder, medico-magical or plant shadow were seldom mentioned by the informants.

### Use of *C. albidum* for food, folk medicine and wood

Results revealed a high consensus degree of *C. albidum* use as food among the informants (FL = 100%). The fleshy pulp of the fruits is widely eaten by the local populations. The pulp can taste either very sweet or sour. Locally, the variation of the fruit exocarp color is said to be correlated with the pulp taste. The exocarp of the sweet fruits was reported to be yellow while that of the sour ones was thought to have a mixture of yellow and green colours when mature. However, no significant differences were found between gender (*χ*^2^ = 0.108; P = 0.742; df = 1) or ethnic groups (*χ*^2^ = 0.011; P = 0.917; df = 1) for the fruit taste. A significant difference was observed between age groups (*χ*^2^ = 28.895; P < 0.001; df = 2) regarding the fruit taste. Nearly 100% of the young people preferred the sweet fruit taste suggesting a local preference of young people to sweetness taste.

**Figure 4:**
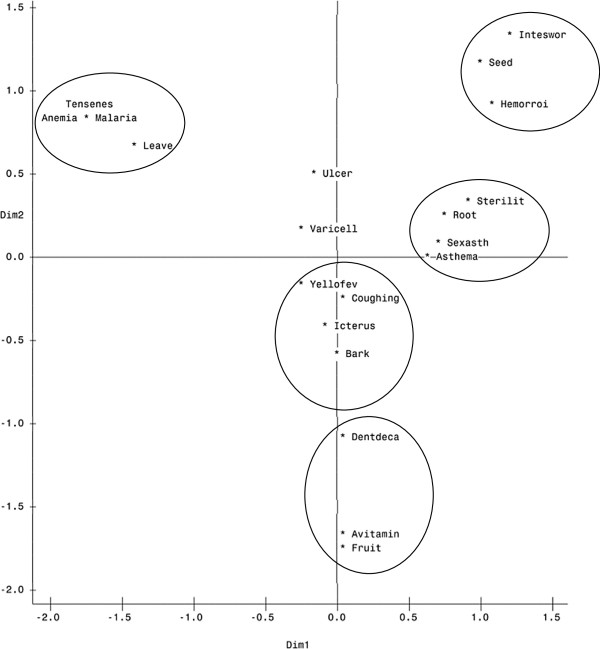
**Correspondence Analysis (Cumulative eigen value for Dim2 & Dim1 = 68.3%).** Legend Malaria = malaria; Asthema = Asthema; Anemia = Anemia; Ulcer = Ulcer, Tensenes = Blood pressure; Yellofev = Yellow fever; Coughing = Coughing; Icterus = Icterus Sterilit = Sterility; Sexual asthenia = Sexasth; Avitamin = Avitaminosis; Varicell = Varicella; Dentdeca = Dental decay; Intesworm = Intestinal worms; Hemorroi = hemorrhoid; Fruit = Fruit; Leave = leaves; Bark = bark; Root = root; Seed = seeds.

Different *C. albidum* plant parts were involved in folk medicine. Results showed that the bark of the species was the most used part by the informants for ailments treatment (FL = 28.04%), followed by the leaves (FL = 22.96%), the roots (FL = 9.57%), the seeds (FL = 5.32%) and the fruits (FL = 1.06%). Fifteen ailments were reported to be treated with *C. albidum* (Table 2). The correspondence analysis (15 ailments X 5 plant parts) revealed a strong relationship between the different plant parts and the type of ailment treated (Figure 4). The leaves were frequently used to treat malaria, blood pressure and anemia. The roots were involved in the treatment of sterility, sexual asthenia and asthma; while seeds were mostly used to treat intestinal worms and hemorrhoid. The bark was used against cough, icterus, yellow fever and the fruits against avitaminosis and the dental decay. Despite this link between plant part and disease, ulcer and varicella treatment were not related to a specific plant part (Figure 4).

**Table 2:** **Method of transformation and processing of different plant part, forms of uses, ailment treat and fidelity level of uses of*C. albidum***

**Plant parts**	**Method of transformation and processing**	**Form of use**	**Aliment treated**	**Fidelity level (%)**
**Leaves**	Boil leaves with cut fruits of *Citrus limon*	Drink the liquid of decoction thrice per day until recovered	Malaria	64.5
	Boil leaves with *Heliotropium indicum* plant	Drink the decoction	Blood pressure	20.8
	Boil dry leaves	Drink the decoction	Anaemia	35.2
	Boil leaves	Drink the decoction	Ulcer	6.6
**Seeds**	Dry and transform the seeds in flour	Drink a little quantity of the flour with water	Intestinal worms	8.5
	Grind the seeds and add palm oil (locally named *tchocho*)	Pass the oil to the anus	Haemorrhoids	17.2
**Roots**	Boil the roots with the leaves	Drink the decoction	Smallpox	4.1
	Boil the roots	Drink the decoction	Asthma	38.3
	Carve up the roots and add fermented water from maize flour or white wine	Drink the maceration	Sterility and sexual weakness	42.6
**Bark**	- Boil the bark with the immature fruit	Drink the decoction	Cough	58.3
	- Boil the bark with Shea butter			
	Boil the bark with leaves and roots of *Cocos nucifera*	Drink the decoction in morning and in the afternoon	Yellow fever	17.5
	Boil the bark	Drink the decoction	Icterus	26.9
	Dry and transform the bark in flour	Add a spoon of the flour in a porridge and drink	Avitaminosis	11.7
	Boil the bark	Drink the decoction	Dental decay	5.4
**Fruit**	Fresh fruit	Eat directly the fresh fruit	Avitaminosis	85.3
	Cut up the immature fruit and add alcohol	Make a gargling with the maceration	Dental decay	16.2

**Figure 5:**
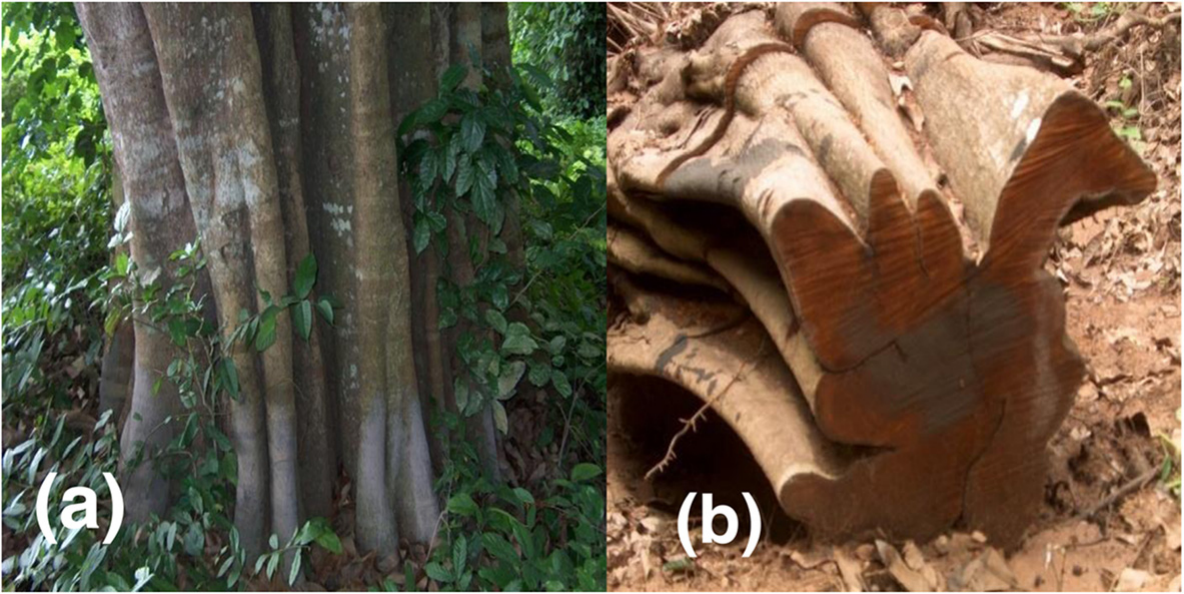
***Chrysophyllum albidum*bole showing its fluted structure.a** = stand tree of *Chrysophyllum albidum*; **b** = logged tree of *Chrysophyllum albidum*.

With respect to the wood of *C. albidum***,** a high proportion (91.5%) of the informants considered its wood as of poor technological quality. It was reported that the bole of the species is fluted and is not suitable for furniture manufacturing (Figure 5). About 84.04% of the informants considered the wood to be good for firewood. However, during the fieldwork, it was noticed that *C. albidum* is rarely cut down for the purpose of firewood. This suggests that that tree owners of *C. albidum* prefer to use the species for its most profitable value (fruit purpose/ailment healing) as frequently argued by informants.

### Others use forms of *C. albidum* and local considerations

Besides the common uses (food, medicine, wood), *C. albidum* leaves were occasionally used for fodder. Rotten or damaged fruits were also used to feed pigs. The species was also used in traditional rituals and was reported to have medico-magical properties. It should be noted that the information regarding *C. albidum* magic properties was considered sacred by the informants and could not be communicated to non-initiated (the interviewers). Some informants stated that the species was used for life renewing by old people and patients who were close to death. It was also mentioned to be used to chase bad spirits.

According to local socio-cultural considerations, there were several taboos regarding the species cultivation. Informants considered that *C. albidum* trees can only be cultivated by older people in order to avoid early death of a young person who would attempt to propagate the species from seed.

### Knowledge variation between gender, age and ethnic groups

No significant differences were observed between ethnic groups in the different use categories of *C. albidum* (Table 3) showing that both ethnic groups used the species almost in the same way. However, the medicine and firewood use significantly depended on gender. Women quoted nine ailments for which *C. albidum* was involved as remedy while men cited thirteen ailments. A total of eight ailments were reported by both genders.

**Table 3:** **Use value of*C. albidum*according to ethnic group, gender and age category**

	**Ethnic group**				**Gender**				**Age category**				
	**Aizo**	**Fon**	**U**	**P**	**Women**	**Men**	**U**	**P**	**Young**	**Adult**	**Old**	**H**	**P**
**Medicinal**	0.81	0.56	3768	0.27	0.26	0.96	3752	0.00^***^	0.05	1.19	1.53	50.99	0.00^***^
**Firewood**	0.95	1.22	3633	0.24	1.26	0.87	3092	0.01^**^	0.94	0.97	1.00	0.11	0.95
**Timber**	0.12	0.01	3736	0.90	0.09	0.11	3394	0.85	0.05	0.11	0.21	0.97	0.62
**Food**	2.96	2.90	3752	0.75	2.96	2.97	3385	0.91	2.95	2.97	3	0.1	0.95
**OUV**	4.84	4.69			4.57	4.91			3.99	5.24	5.74		

OUV= Overall use value, U = Mann–Whitney Statistics, H = Kruskal-Wallis statistics, P = Level of significance;

** *P* < 0.01, *** *P <* 0.001.

Similarly, significant differences were observed between age classes regarding the medicinal uses of *C. albidum*. Young informants knew little about the medicinal use of the species while old people considered the species as a highly valuable taxon for medicine. Lastly, informants of different age classes presented equal knowledge on *C. albidum* regarding its use as timber, food and firewood.

Overall, the ethno-botanical use value of *C. albidum* was almost similar for the two ethnic groups (OUV_fon_ = 4.84 and OUV_aizo_= 4.69) and for both genders (OUV_Women_= 4.57 and OUV_Men_= 4.91). Regarding age groups, the ethno-botanical use value seemed to be lower for the young (OUV_young_ =3.99) than for the adults (OUV_adult_ = 5.24) and old people (OUV_old_ = 5.74).

## Discussion

### Utilization and use categories of *C. albidum*

This study highlights the multipurpose character of *Chrysophyllum albidum* in Benin. Four use categories were determined for the species. Three use categories (food, medicinal and firewood) emerged as having a high consensus degree among the informants in the region. Our findings are consistent with previous studies which reported rich and diverse array of uses of *C. albidum* trees [15,33,34]. For instance the species was mentioned to be highly valued in traditional medicine and its fruits widely consumed in Nigeria [19,35] as it was observed in our study area. While Adu-Boadu [33] mentioned the medicinal value and nutritive value of various parts of *C. albidum*, Boateng and Yeboah [34] highlighted the trade value of this species’ fruits for food purpose in Ghana.

With respect to the different plant parts, results showed that the fruit (food property of the pulp) of the species was the most valuable non-timber forest product while the bark and leaves were used in folk medicine, which is in agreement with results from Edem *et al.*[36] and Odugbemi *et al.*[19]. The nutritional value of *C. albidum* was already assessed by Edem *et al.*[36] who showed that the pulp of the fruit contains 8.8% of protein; 15.1% of lipid, 68.7% of carbohydrate, 3.4% of ash, and 4.0% of crude fiber. This high nutritional value might justify the relative importance of the fruit consumption by local populations and therefore, its presence in the traditional agroforestry systems in the studied area. However, food processing initiatives of the fruits are rare in Benin Republic while in other countries like Nigeria and Ghana, the transformation of these fruits for table jelly, drinks and others are growing initiatives [15,16,37]. Previous studies also reported that the seeds of *C. albidum* are rich in linoleic (38.4%) and oleic (29.6%) acids and could be used in free fatty acid production [38,39]. However this use form was not reported in our study area and the seeds are thrown away probably due to lack information on the potential of the seeds, and lack of facilities nearby to process it. Despite these potentials, *C. albidum* is less exploited in the southern Benin than in Ghana and Nigeria and less incorporated to the commercial agricultural production system. The use of *C. albidum* remains traditional and the species underutilized.

With regard to medicinal use, our findings revealed the specificity of the different plant parts in ailment curing. The bark of the species was locally used for healing coughing, icterus and yellow fever while the leaves were employed to treat malaria, high blood pressure and anemia. In southern Nigeria, it was reported that the bark was used to treat the yellow fever and malaria, while the leaves were used as an emollient and for the treatment of skin eruption, stomachache and diarrhea [40,41]. In this study, we reported the use of *C. albidum* roots in traditional gynecology (treatment of sterility, sexual weakness) while Okunomo and Egho [42] reported the fruits for the same purpose. This difference in *C. albidum* plant part in traditional gynecology highlights the variability of ethno-knowledge between ethnic groups. These results emphasize the importance of undertaking biochemical analysis of the different plant parts in order to confirm or to infirm the traditional medicine use of the species. Yet, studies carried out in Nigeria showed that the leaf extract of *C. albidum* contained antiplatelet and hypoglycemic compounds and could be employed in the treatment of myocardial infarction and diabetes mellitus respectively [43]. The methanolic bark extract of *C. albidum* contains anti-plasmodial substances and could be used in treating malaria [44]. Moreover, it has also been reported that methanolic extracts from *C. albidum* leaves presented strong antibacterial activity against common bacteria such as *Escherichia coli* T. Escherich, *Salmonella typhimurium* Loeffler, *Staphylococcus aureus* Rosenbach [45]. However, all these reported proprieties abovementioned remain laboratory findings (since they were only tested on rodents) and need to be further investigated. Nonetheless these are important findings for future biochemical and pharmacology studies of the species for medicinal properties.

Although the species was reported to be highly valued as timber in another regions (Uganda for instance) due to the appreciable physical and mechanical properties of its woods [46], this was not the case in the southern Benin (Plateau of Allada). The species was generally only cut down in the case of other land use such as house building or road construction. In fact, the bole of *C. albidum* often presents a network of fissures and this was reported locally as a major impediment for the species wood used as timber. Thus, contrary to many other trees species, *C. albidum* did not appear to be threatened by logging. However, it should be noted that this species’ habitat has been considerably reduced due to the growing urbanization in the southern Benin.

### *Chrysophyllum albidum* use knowledge variation

Our findings showed significant differences on the species’ use value for medicine between age groups and genders. Previous studies on others plant species came to the same conclusion and stated the uneven distribution of indigenous knowledge for local plant use [20,23,47]. However, in this study we did not find ethnic differences in use value of *C. albidum* which is contrary to other studies on NTFPs [4,5,21,48-51]. This result may be related to the cultural link between the two investigated ethnic groups. Historically these two ethnic groups belong to the same cultural group “Alladanou”. Even though the Fon ethnic group migrated to the “Plateau of Abomey” in the centre part of Benin, the two groups still cohabit today on the “Plateau of Allada”. Nowadays, they are commonly mixed and share most folk knowledge, traditional value and rituals regarding many practices. Therefore, as far as ethnobotanical knowledge is concerned, we deduced that the cultural origin might be an important factor to take into account in medicinal plants value assessment. However further studies are needed to confirm that assertion not only in the specific case of *C. albidum* but also for many other multipurpose plant species.

The significant increasing medicinal use value of *C. albidum* with increasing informant age confirms the assertion of increasing ethno-medicine knowledge of plant species with age [52-54]. Because of this age related knowledge, there could be a long-term loss of medicinal knowledge. The disappearance of the current "old generation" might involve the loss of folk medicine on *C. albidum* since young informants mostly rely on modern medicine. Instead of being complementary, modern medicine appears sometimes as an impediment to the development of folk medicine [55,56]. So far, intensive and continued research on ethnomedicinal value of plants is needed not only for *C. albidum* but for many other species in order to document and to preserve the traditional knowledge of local population other the time.

The current knowledge of *C. albidum* (mainly medicinal and alimentary use) provided by the informants in this study can be regarded as an opportunity for its conservation and cultivation for livelihood enhancement. Moreover, the large potentialities (pulp and seeds use in food industry, plant parts use in pharmaceutical laboratory) of the species and which are not fully exploited in the studied area may militate in favor of its conservation and promotion. The study also demonstrated that, the local current use of the species is not destructive (i.e. tree cutting for timber is scarce). The main concern of the species’ stands conservation is related to its habitat destruction. Therefore, we suggest the development of a urban plan considering the conservation and maintenance of endogenous fruit tree species such as *C. albidum*. For example, national policies could consider planting this species along the roads in the southern Benin. In order to fulfill this suggestion, research on the species' reproduction, growth and survival, and on the fruit phenotypic characterization should be carried out to provide baseline data for the selection and planting of superior individuals.

## Conclusion

This study highlighted the multipurpose nature of *Chrysophyllum albidum*. Its fleshy fruits are widely consumed and the different plant parts are used in folk medicine to treat several diseases and disorders. Although the fruits of this species contribute to improve health, nutrition, food security and income of the local communities, the species could be further exploited in the region. In addition, *C. albidum* is threatened by habitat loss. Therefore, it is important to develop sustainable strategies for the species conservation. One option to explore is to plant this species around urban areas together with the protection of the current existing specimens. In order to facilitate its cultivation more information on agronomic, genetic and economic aspects should be further studied. Urban forestry based on *C. albidum* could help promote the species in other sub-Saharan region where the species stand is facing decline due to cities expansion.

## Competing interests

The authors declare that they have no competing interests.

## Authors' contributions

LGH and TOL were involved in the study design, proposal writing for data collection and, data analysis. They wrote the first draft of this manuscript. LESA and FGHG were involved in data collection (field work) and data computing. BS was supervisor of the study; he read and contributed to improve this manuscript. All authors read and approved the final manuscript.
